# Operationalizing fair AI in healthcare: a scoping review of bias mitigation, monitoring, auditing, and governance strategies

**DOI:** 10.3389/fdgth.2026.1888744

**Published:** 2026-10-13

**Authors:** Ana Teresa Rebocho, Sara Silva Marques, João Pedro Santos, Cátia Sousa Pinto

**Affiliations:** Global Digital Health and International Affairs Unit, SPMS - Shared Services of the Ministry of Health, E. P. E., Lisbon, Portugal

**Keywords:** artificial intelligence, auditing, bias mitigation, fairness, governance, healthcare

## Abstract

**Objectives:**

This study explores the challenges of deploying fair artificial intelligence (AI) solutions for health applications, identifying bias detection and mitigation strategies, along with the policies, governance, and monitoring mechanisms necessary for operationalizing fair AI in healthcare.

**Methods:**

A scoping review of scientific and grey/regulatory literature was conducted following PRISMA-ScR guidelines. Searches were performed across academic databases and other relevant sources from January 2022 to January 2026. Eligible studies reported AI fairness challenges, bias mitigation strategies, and perspectives on AI governance, auditing and continuous monitoring.

**Results:**

The 66 documents included address the challenges in operationalizing fairness throughout the AI lifecycle, including examples of fairness monitoring methodologies considering the regulatory landscape. Barriers and gaps were identified to explore how bias mitigation strategies could be harmonized. The discussion provides an in-depth analysis of fairness-aware measures identified in the healthcare AI literature, while also evaluating the importance of continuous monitoring, AI auditing and governance structures. This resulted in a set of recommendations towards fairness to implement throughout the AI lifecycle for healthcare solutions.

**Conclusion:**

This study provides a set of recommendations for enabling AI fairness in healthcare. These include improved data representativeness, fairness-aware measures, auditing and governance frameworks. Their successful implementation will require interdisciplinary collaboration among multidisciplinary stakeholders and coordination between governance bodies to address the fairness concerns that the rapid advancements of AI bring and to ensure its trustworthiness.

## 1 Introduction

The rapid development of AI technologies for healthcare brings with it an increased awareness of ethical and fairness concerns. In recent years, growing efforts have focused on effective strategies that place ethical, social and trustworthy foundations at the centre of responsible AI deployment and integration in healthcare (1, 2).

Fairness is a fundamental aspect of healthcare delivery, based on ethical principles that encompass distributive justice and societal dimensions, aiming equal allocation of resources, opportunities, and outputs without discrimination (3). Similarly, AI fairness in healthcare encompasses the ethical and equitable distribution of these resources and unbiased decision-making across population groups (4).

Despite the benefits surrounding AI health technologies, concerns remain regarding their misuse or unethical application. Understanding, measuring and mitigating biases is essential to strengthen the ethical deployment of AI in real-world healthcare, ensuring equitable outputs delivery while fostering trust and acceptance of these solutions among patients and health practitioners (5).

In this context, bias refers to systematic errors or deviations that may introduce or exacerbate healthcare disparities. This bias may result from flaws and decisions taken at any point of the AI lifecycle (6, 7), namely during modeĺs design, development, deployment and post-deployment monitoring (Supplementary Figure S1). Biased AI systems can promote or reinforce systemic errors or inequalities in clinical decision-making and access to care, disproportionately affecting underrepresented and under-resourced populations (8–10). Hence, addressing fairness requires a holistic, AI lifecycle-oriented approach (11–13).

A careful and rigorous evaluation of the adequate bias mitigation measures to be applied, throughout the AI lifecycle, must be conducted to avoid the introduction of noise, overfitting and other factors that may impact the accuracy-fairness trade-off (14).

In this context, robust legal and regulatory frameworks that guide the integration of AI in healthcare are needed to address these complex ethical and technical challenges. By providing clear regulatory compliance mechanisms, such frameworks can advance innovation in healthcare through trustworthy and ethical deployment of AI (15).

In the European Union (EU), AI applications for health are primarily governed by the Artificial Intelligence Act (AI Act) (16) and the Medical Device Regulation (MDR) (17). These frameworks establish complementary requirements and obligations, including the use of regulatory sandboxes pre-market and the implementation of post-deployment surveillance strategies (13, 18–20). However, the AI Act does not address specific implementing acts focused on inherent fairness issues. Guidance to support AI developers and providers in integrating fairness and managing trade-offs throughout the AI's lifecycle remains lacking (21). Moreover, ambiguities in certain AI Act requirements create uncertainty for developers, regulators, and the industry (22, 23).

As AI systems move toward real-world deployment in healthcare, fairness provisions must be a priority within AI lifecycle all the way through post-deployment (24). The complexity of AI models, their dynamic learning nature and their sensitivity to contextual changes may compromise the long-term sustainability and fairness of AI systems. These factors can lead to fairness drifts, defined as inequitable changes over time in AI system performances across population subgroups. Accordingly, continuous monitoring is required to identify and address such drifts as they occur (3, 25–27).

Therefore, the establishment of harmonized fairness frameworks for monitoring and auditing AI systems compliance with regulatory requirements throughout their lifecycle, particularly during post-market, is needed. Addressing this gap is pivotal for regulatory bodies that face the dual challenge of keeping up with the introduction of AI technologies in healthcare while supervising their use and implementation in medical facilities (18, 26).

This article aims to explore the challenges associated with the deployment and monitoring of fairness in AI systems for healthcare purposes, focusing on the following research questions:

1. What bias mitigation strategies are reported in the literature to support fair AI in healthcare?
2. How should such systems be monitored and audited in practice?
3. What policies and governance mechanisms are necessary to support the surveillance of fair AI systems in healthcare throughout the AI lifecycle?

A scoping review was conducted to address these research questions, mapping the most frequently reported bias mitigation measures throughout the AI lifecycle and the current auditing practices. Moreover, this scoping review intended to identify the barriers and emergent needs regarding AI fairness that require addressing throughout the AI lifecycle. Finally, to leverage the findings into actionable solutions to guide the future deployment of fair AI in healthcare, recommendations to support the operationalization of fairness are proposed.

## 2 Methods

### 2.1 Data sources and search strategy

An analysis of literature was conducted following Preferred Reporting Items for Systematic Review and Meta-Analyses extension for Scoping Review (PRISMA-ScR) guidelines to answer the above-mentioned research questions. PubMed, Science Direct and Google Scholar academic databases were used to search for studies published between January 2022 to January 2026. The search was conducted between February and March 2026. To ensure alignment between the search strategy and the research questions, keywords related to the main domains of the review were selected and combined using Boolean operators to construct the following search queries (SQs):

SQ1) AI healthcare AND (“bias” OR “fairness” OR “bias mitigation”).
SQ2) AI healthcare AND fairness AND (monitoring or regulatory or audit).

For each SQ, the search results were sorted by relevance, and the first 200 results from each database were evaluated, ensuring a comprehensive examination of the available studies.

Additionally, to ensure a policy-aligned analysis, relevant grey and regulatory literature from international and institutional sources was also incorporated (Supplementary Table S1). The documents from these sources comprised relevant legal frameworks, reports, guidelines, recommendations and informative materials related to AI governance, regulatory oversight and implementation of AI in medical devices, and in health systems. This grey and regulatory literature was searched in additional databases, such as the Eur-Lex, European Commission (EC) and Parliament, European Medicine Agency (EMA), Food and Drug Administration (FDA), and the World Health Organization (WHO). Since these databases offer limited search functionalities and differ in scope, the searches were performed using broader and more adaptable terms: “artificial intelligence” and “lifecycle” and “healthcare” and “bias” and “monitoring” and “regulation” and “authorities” and “surveillance” and “governance” and “ethical” and “medical device” and “recommendation”.

### 2.2 Eligibility criteria

For the scientific literature, only English-language scientific documents subjected to a peer-review process were evaluated. Studies for which the full text was unavailable, as well as citation-only entries, were excluded. To focus the findings on improving AI fairness in healthcare, studies unrelated to AI applications for healthcare were not considered. Finally, the identified references were screened according to the inclusion criteria, which focused on studies covering (1) fairness across the AI lifecycle or stages, (2) bias mitigation strategies, and (3) governance, auditing and continuous monitoring recommendations for fairness. Exclusion criteria included studies that did not relate to the stages of deployment and implementation of fair AI, that lacked deep analysis on perspectives for bias mitigation strategies, or studies that did not address relevant monitoring and governance mechanisms for the development and use of fair AI in healthcare.

For grey and regulatory literature, only official documents and informative materials issued by recognized international organizations, regulatory authorities, and governmental institutions were assessed for inclusion and considering their relevance to the scope of the study. Eligible documents included regulations, guidance documents, recommendations, technical reports, and informative materials addressing the governance, regulation, lifecycle management, monitoring, or ethical deployment of AI in healthcare and AI-enable medical devices. From these, documents that addressed one or more of the following topics (1) stages of the AI lifecycle, (2) bias identification or mitigation strategies, and (3) governance, regulatory oversight, market surveillance, or continuous monitoring of AI systems in healthcare were considered. Documents unrelated to healthcare AI, duplicate publications, non-English documents, or documents without full text access were excluded.

### 2.3 Data selection and extraction process

The results retrieved from the databases searched were exported to CSV format. Records not published in English or with unavailable full text were removed.

Duplicate records were identified using titles of articles and studies and subsequently removed. The deduplication process was independently conducted by two authors. The first author (ATR) performed the process manually in Microsoft Excel, while a second author (JPS) conducted the same deduplication process using Python to verify consistency and accuracy of the results. Any discrepancies were reviewed and resolved through discussion.

Subsequently, the titles and abstracts of the remaining articles were independently screened by three reviewers (ATR, JPS and SSM) against the predefined inclusion and exclusion criteria to detect subject relevance to the research objectives. Any disagreements regarding eligibility were resolved through discussion and consensus.

Following title and abstracts screening, the full text of the resulting articles and grey literature documents were analysed based on the eligibility criteria. Full text screening enabled a detailed evaluation of the study relevance to the research questions, resulting in the exclusion of articles that were not substantially aligned with the study objectives. Discussion between the three reviewers and agreement were used to settle any disagreements about eligibility.

### 2.4 Data synthesis and analysis

Eligible studies were analysed and their findings were organized under thematic categories for data synthesis, aligned with the scoping review's research questions. These categories were used to structure the analysis of the findings, by identifying the main practices reported in literature, covering (1) fairness across the AI lifecycle, (2) bias mitigation strategies, and (3) governance, auditing and continuous monitoring reports.

The results were synthesized using a descriptive and narrative approach. The first allowed to map the features of the included studies, while the narrative approach allowed a comparative interpretation rather than a quantitative meta-analysis.

Based on the analysis of these findings, valuable insights were derived to construct recommendations addressing the barriers identified towards the deployment and operationalization of fair AI systems in healthcare.

### 2.5 Limitations

This study presents several limitations. First, although the databases allowed the identification of a broad range of relevant studies, the search may not have captured all publications on the topics addressed by the research questions. The decision to screen only the first 200 results from each database per search query may have excluded relevant studies appearing lower in the results. On further, Google Scholar results ranking according to relevance are less reproducible than those of academic databases.

Secondly, our literature search strategy was intentionally focused on studies addressing AI in healthcare in relation to fairness, bias mitigation, continuous monitoring, governance frameworks, regulation, and audits. While this focus was designed to capture the core challenges within the defined scope of the review, it may have overlooked studies that use alternative terminologies.

Thirdly, scientific literature included in the search was limited to English-language peer-reviewed and full text open access articles. This may have excluded relevant non-English studies in other forms of academic output, and subscription-restricted publications.

The institutional and regulatory sources consulted were selected to provide complementary policy perspectives relevant to the scope of the analysis. Sources such as WHO and FDA provided documents with a global governance perspective and non-EU regulatory based system vision, respectively. Nevertheless, due to the predominance and use of European institutional sources, the analysis may introduce partly EU-centric regulatory framing, potentially underrepresenting alternative approaches from other regions. Additionally, no formal critical appraisal of methodological quality or risk of bias was conducted in the included literature, which might limit their robustness.

Finally, this study reflects the evidence available at the time of the analysis. Given the rapid evolution of the field, including technical, governance and regulatory developments, future research should update and expand these findings as new evidence emerges.

## 3 Results

### 3.1 Description of the included studies

Figure 1 provides a visual representation of the search results and the process for studies selection. From the search conducted in databases and registers, a total of 1030 studies were identified, 600 corresponding to SQ1 and 430 to SQ2 (as only 30 studies resulted from the PubMED search for SQ2). From these, 132 duplicates and 178 records (non-English language and unavailable full text) were removed. The remaining 720 studies were thoroughly examined and assessed for screening. From this process 176 were sought for retrieval and assessed for eligibility criteria.

**Figure 1:**
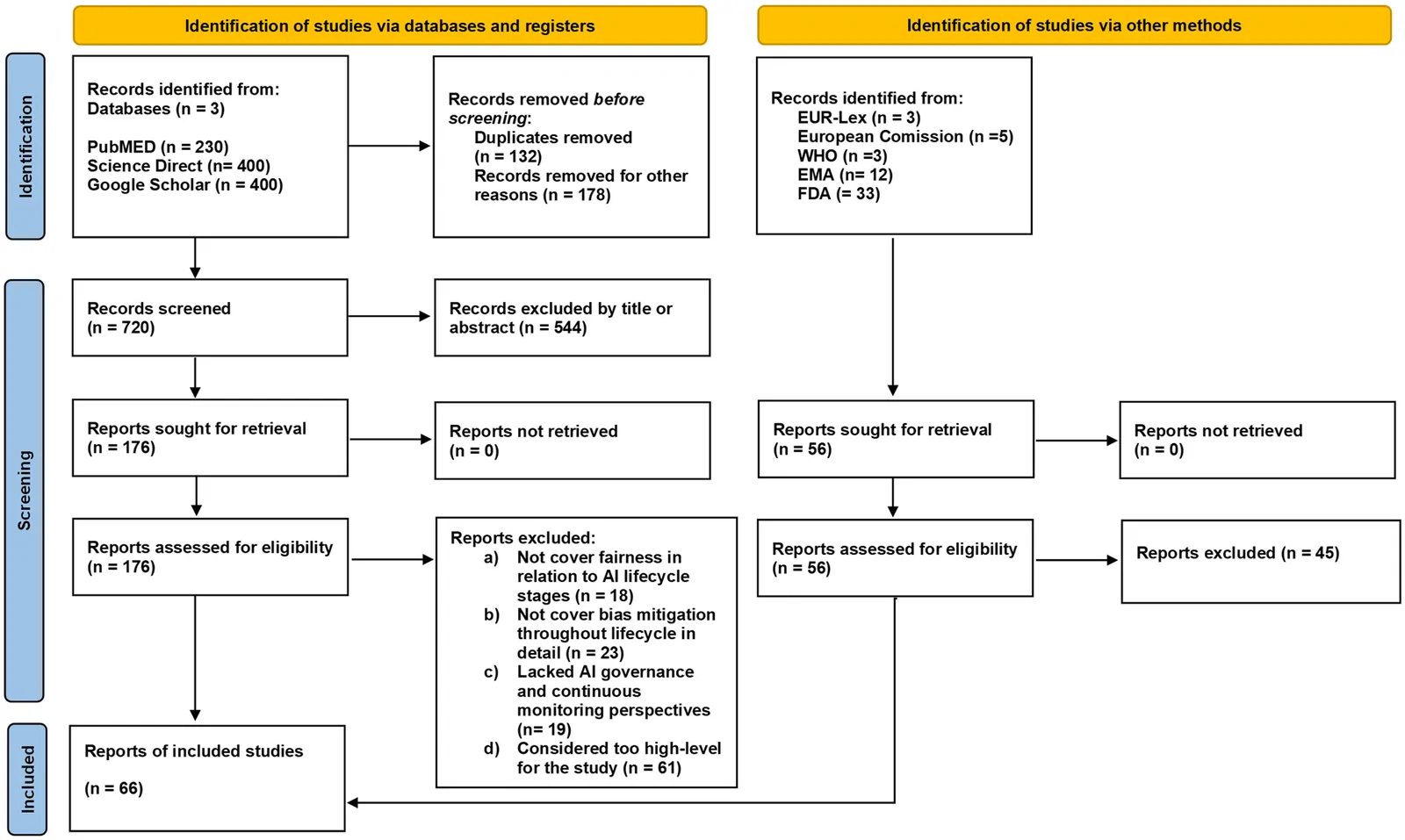
PRISMA flow diagram.

Concerning the grey and regulatory literature, 56 documents were identified through searches of official sources, including EUR-Lex (*n* = 3), EC (*n* = 5), WHO (*n* = 3), EMA (*n* = 12), and the FDA (*n* = 33). The documents were retrieved and assessed for predefined eligibility criteria, during which 45 documents were excluded, as they did not address AI lifecycle, bias mitigation approaches, data-related considerations important to healthcare AI, or governance and continuous monitoring mechanisms. Therefore, from this assessment, 11 grey and regulatory documents were included.

Ultimately, a total of 66 studies (Figure 1), 55 from databases and registries and 11 from grey literature, were included in this study, which addressed the bias mitigation strategies most frequently reported across the included sources, and explored AI auditing frameworks and governance mechanisms that support fairness monitoring throughout the AI lifecycle.

### 3.2 Overview of the included studies

The individual studies were assessed and assigned according to the thematic findings aligned with the scope of the review. Table 1 outlines the distribution of the 66 included studies across the following thematic findings: fairness measures, data representativeness, audits, continuous monitoring, and AI governance. As categories were not mutually exclusive, studies addressing multiple topics were assigned to more than one category.

**Table 1:** Distribution of the included studies across thematic review findings.

Thematic findings	n (%)	Included studies
Data representativeness	12.1	(14, 16, 20, 35–38, 54)
Audits	25.8	(3, 15, 26, 37, 49, 50, 56, 58–61, 65, 66, 70, 71, 79, 81)
Fairness measures	34.8	(9, 11, 14, 30, 31, 33, 34, 40–51, 62–64, 67)
AI governance	25.8	(13, 27–29, 37, 49, 52, 57, 69–74, 76, 77, 81)
Continuous monitoring	30.3	(16, 17, 32, 37, 39, 50, 53–56, 58, 68, 75–82)

As shown in Table 1, 34.8% (*n* = 23) of the 66 included studies addressed fairness measures, including pre-processing techniques, fairness metrics and algorithmic testing, all aimed at mitigating bias across the different stages of the AI lifecycle. Moreover, data representativeness was address by 12.1% (*n* = 8) of the studies, particularly regarding the use of the representative and unbiased data during model development and updates, including institutional guidance. Audits were addressed by 25.8% (*n* = 17) of the studies. In this study, auditing refers to the structured assessment of AI systems against several criteria such as bias identification and mitigation, accuracy and regulatory compliance. The findings highlight audits as a key strategy for evaluating fairness, defining audit procedures (i.e., what should be assessed, by whom and when) and promoting accountability to support trustworthy AI. Similarly, 30.3% (*n* = 20) of the studies emphasized the importance of continuous monitoring to evaluate AI system performance, mitigate emerging biases and ensure fairness is maintained over time. Furthermore, 25.8% (*n* = 17) of the included studies highlighted the importance of establishing practical and cohesive AI governance structure, with recent institutional reports approaching the necessity of strategic and oversight implementation for AI in health (28, 29).

These thematic review findings directly map onto the research questions guiding this review.

## 4 Operationalizing fair AI in healthcare

In line with the methodology outlined in Section 2, this section charts the practices, challenges, and gaps reported across the included studies regarding the operationalization of fair AI in healthcare.

### 4.1 Harmonization of fairness-aware measures

Over the years, several fairness and bias mitigation approaches have been proposed for application across the different stages and processes of the AI lifecycle (Supplementary Figure S1). These approaches range from data and model level interventions, as well as fairness assessment and monitoring, from design through post-deployment monitoring stages (11, 30, 31). However, operational challenges remain due to problems with biased design choices, data representativeness, the selection and interpretation of fairness metrics, and the trade-off between fairness and model accuracy (32, 33).

Figure 2 synthesizes the main fairness and bias mitigation strategies identified across the included studies and discussed in the present section.

**Figure 2:**
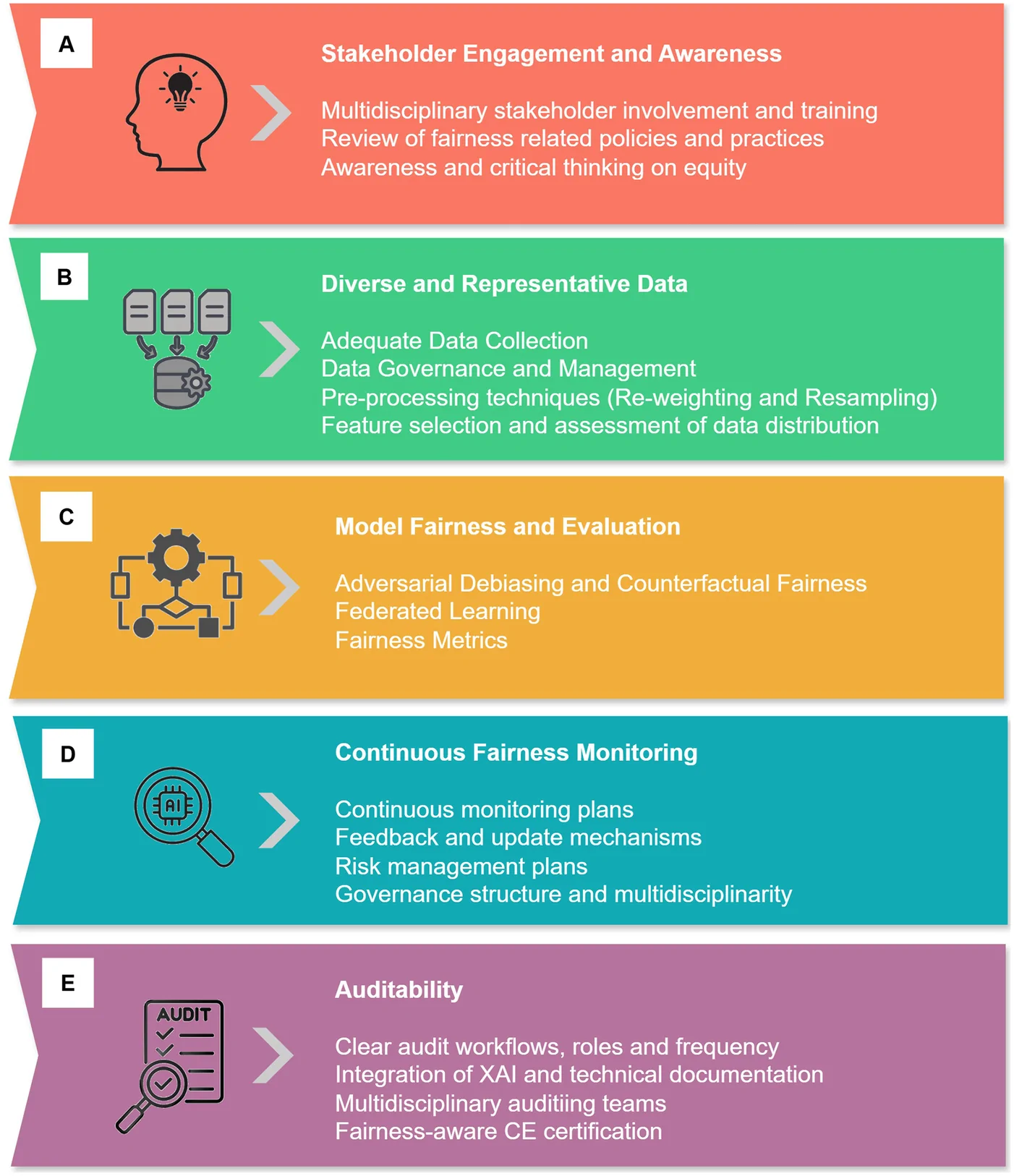
Bias mitigation strategies for the deployment of fair AI for healthcare.

Fairness monitoring and practices should start in early stages of the AI development lifecycle, with the structured inclusion of a multidisciplinary group already in the AI design process, ensuring that appropriate training exists on those engaged so that equity considerations are embedded from the outset (Figure 2A). This should be complemented with review of established policies and routines, to promote awareness and critical reflection on the implications of design choices on different populations and, consequently, on fairness (11, 34).

Unbalanced, incomplete, or skewed health datasets, resulting from the systematically exclusion or underrepresentation of certain communities, as well as from insufficient subgroup sample sizes, are common (34–36). These often represent the only data available for training algorithms, which may decrease models' performance across population subgroups and increase inequities. Thus, improving data collection practices across different settings, locations and demographics, to ensure adequate representativeness of the target population during AI development stage is paramount to promote fairness (11, 20) (Figure 2B). This can also be achieved through enhanced community engagement, partnerships during the AI design phases, and careful selection of features (34).

As AI success depends on high-quality and secure data, thorough data management and governance measures throughout data lifecycle (i.e., collection, storage, access and sharing) must ensure data is current and representative (Figure 2B) (37). WHO and FDA have emphasized bias mitigation through the use of representative data and robust health data management and governance frameworks, ensuring the development, testing and monitor of AI systems with large, high-quality, and ethical datasets (38, 39). Similarly, the AI Act and the MDR highlight the importance of examining and providing representative data of the target population. Under these, manufacturers are expected to implement data governance and management, as well as bias mitigation measures (20). These requirements apply to an AI system intended to be used as a medical device or as a safety component of one, and are subjected to third-party conformity assessment by a notified body, classifying as high-risk AI system, under Art.6 of the AI Act (16, 20).

Establishing robust data governance measures at early stages of the AI pipeline is also paramount to ensure data representativeness (37). Therefore, incorporating a multidisciplinary board encompassing AI developers, clinicians and legal and ethical experts to review the representativeness and quality of datasets collected, and to provide fairness guidance throughout the AI design, while ensuring compliance with data privacy and secure access regulatory requirements is pivotal (37, 40).

The adequate curation of real-world datasets is also essential to address imbalanced data distributions, such as physical characteristics, demographics and attributes (36).

In addition to the previously described upstream strategies, it is crucial to implement technical bias mitigation measures further ahead. Re-weighting datasets, resampling their size (e.g., by generating synthetic data), feature selection and distribution are amongst the most used fairness-aware techniques for pre-processing during data preparation at AI development stage (Figure 2B) (41–44).

The selection and success of each approach depend on the characteristics of the dataset and the specific fairness issue being addressed. Re-weighting allows developers to modify the weights assigned to the instances of different groups within the training samples to prevent imbalance. On the other hand, resampling adjusts the size and distribution of a dataset by increasing (oversampling) or decreasing (under-sampling) the representation of certain groups without altering dataset´s inherent characteristics (14, 44). Oversampling is the resampling method most described in literature (11, 44).

Within oversampling methods, the Synthetic Minority Oversampling Technique (SMOTE) generates synthetic samples by interpolating between minority-class instances to improve balance. However, this may come at the cost of producing unrealistic data points that may not reflect real-world representation (11, 41, 42).

Synthetic data generation methods are often referred to as strategies to mitigate bias as artificial data that mimics the statistical properties and patterns of real-world data without compromising data privacy (41). Generative Adversarial Networks (GANs) and Variational Autoencoders (VAEs) are examples of these synthetic data generation methods (41, 43). Each presents its own trade-offs depending on data type and target application.

GANs are usually applied to generate realistic synthetic medical imaging, such as x-rays, MRIs and CT scans, while comparing it to real-world data. This enables the creation of realistic synthetic datasets used to train diagnostic systems. Nevertheless, the use of small datasets may decrease the representativeness of outputs. Also, GANs requires high computational demands and high-quality data to produce good results (41), and training the models is often considered challenging (9). In comparison, VAEs create synthetic data without exposing sensitive information by encoding real-world datasets, such Electronic Health Records (EHRs), into a lower-dimensional latent representation. Although VAEs' output achieve lower realism than those obtained by GANs, VAEs are usually easier to train and tend to provide greater data samples diversity (41, 45).

Likewise, several techniques can be applied during model training, validation and testing to mitigate biases still during the development stage of the AI lifecycle (Figure 2) (30, 46).

Federated learning provides model access to diverse datasets distributed across multiple institutions through a decentralized and collaborative approach (11, 41). During model development and training stages, healthcare institutions share trained model parameters, which are subsequently aggregated to generate a global model without requiring the exchange of raw data from institutional boundaries (11, 41). This approach facilitates privacy preservation and minimises the logistical resources associated with centralised data integration and interoperability challenges (11, 41). However, the quality and complexity of real-world data can significantly impact local pre-processing stages and, consequently, model performance, as the nature of federated learning restricts centralized data harmonization and quality assessment across institutions (11, 41).

Additionally, adversarial debiasing has been reported as an approach for reducing algorithmic bias during model development and training (Figure 2C). It involves two neural networks, in which one network, “the predictor”, learns to predict the outcome, and the second, an “adversary,” attempts to infer sensitive attributes from the predictor's learned representations or outputs (44). This process seeks to balance predictor accuracy while reducing its reliance on sensitive attributes (e.g., race or gender) or related proxies which may contribute to biased predictions (14). However, this technique effectiveness is highly context-dependent, and may introduce fairness and accuracy trade-offs (11). A study has demonstrated that the efficiency of adversarial debiasing depends on dataset characteristics, including data balance, representation, and the relationship between sensitive attributes and predictive features. Thus, generalizability across clinical contexts and different population remains a limitation, requiring a careful evaluation of its application (11, 14).

On the other hand, counterfactual fairness can also be applied during the development stage. Based on causal modelling, counterfactual fairness evaluates whether predictions remain consistent upon alterations in sensitive attributes (e.g., race, gender, age), assessing whether these may causally influence in the AI decision-making processes (Figure 2C). However, its application depends on the identification of causality in the decision-making process, which requires further validation for its applicability in real-world settings (47).

During model validation and testing, fairness metrics are commonly applied to measure whether the model's performance differs across patient populations (Figure 2C) (47, 48). These metrics quantify disparities related to sensitive attributes by assessing differences in statistical outcomes, such as error rates, true and false positives, predictive values, or overall accuracy. They provide insights if the AI systems perform differently across patient groups while supporting the identification of potential algorithmic bias (5, 11, 31).

Frequently reported metrics include predictive parity, equality of opportunity, delta accuracy, equalized odds, and demographic parity. Nevertheless, literature highlights that these metrics represent distinct and sometimes competing notions of fairness (11, 31, 47, 48). For instance, equality of opportunity assesses whether false negative rates are comparable across groups, predictive parity evaluates whether positive predictions have similar predictive values between protected and privileged groups, and demographic parity examines whether different groups have similar probabilities of receiving a positive model prediction (11, 31).

Thus, the selection of appropriate fairness metrics should be tailored to the specific healthcare application and guided by its clinical context (31, 47, 48). Moreover, providing an ethical definition of these metrics is crucial throughout the AI lifecycle. Nevertheless, their validity relies on stakeholders’ perception of the outcomes (49).

A study on developers' perspectives reported that fairness practices should be continuous and are context-dependent, since a one-size-fits-all approach is often ineffective (50). For example, developers might use resampling techniques to balance data and counterfactual analysis to assess fairness in decision-making across populations, while ethicists may provide guidance during the AI lifecycle for continuous alignment with social and ethical values (40). Therefore, it is essential to engage multidisciplinary stakeholders. Their involvement would facilitate the inclusion of diverse viewpoints and perspectives during the co-creation of recommendations and standards for trustworthy AI systems (Figure 2A) (51).

Despite the importance of fairness testing and post-prediction analysis (i.e., analysis of the predictions made by the model) to improve fairness, their implementation is still limited, indicating a gap between their recognized importance and real-world adoption (50). Thus, promoting organizational awareness and establishing support mechanisms, through fairness training, integrating fairness into AI pipelines, and creating collaborative tools for interdisciplinary teams is essential (50).

### 4.2 Governance for fair AI in healthcare

The operationalization of AI fairness presents itself as a multifaceted challenge extending beyond the technical mechanisms, also intertwining organizational practices, regulatory contexts and human expertise, all playing a role throughout the AI lifecycle. This depicts the sociotechnical nature of operationalizing fairness in AI for healthcare (52).

Tackling AI fairness goes beyond applying established fairness-aware measures, requiring a robust AI governance framework. This must entail a set of guidelines and procedures to ensure fairness compliance throughout the AI lifecycle. Such framework must encompass standards for continuous monitoring and evaluation, audit frameworks, data governance and management plans, along with regular assessments of regulatory compliance (37, 53).

#### 4.2.1 Continuous monitoring and auditing

Continuous monitoring of AI systems upon post-deployment is vital to ensure identification and recalibration of systems that experience concept drifts, errors and misuses over time, which can compromise accuracy, safety, and legal compliance (37, 54). Such recalibrations require the establishment of plans for surveillance of fairness (Figure 2D), as advocated by a study on UK healthcare professionals', since biases may emerge from non-representative or incomplete datasets, opaque outputs (53), as well as data drifts (39, 54). Thus, continuously retraining AI models with inclusive and fresh data is of utmost importance to address possible biases, along with the implementation of robust oversight protocols at a post-deployment stage of their lifecycle (53).

EMA and FDA also emphasize the need for continuously monitoring the performance of AI/ML models (54, 55). Continuous monitoring frameworks to actively assess models' performance, with well-defined procedures and metrics must be established, along with risk management plans to proactively correct/update or decommission the model as applicable (55). FDA's recent recommendations propose post-deployment monitoring plans, referring to the ongoing assessment of an AI-enabled medical device in a post-market setting, to support corrective actions or model updates whenever needed towards maintaining its effectiveness and safety throughout time (39, 54).

Similar to what happens in the pharmaceutical sector, the inclusion of fairness validation thresholds for the model outputs, established under the guidance of national level competent authorities, could represent an advantageous mechanism during surveillance and auditing activities (56). Following a risk-based and context-specific approach, these thresholds could serve as a reference criterion for classifying fairness-related misuses, errors and assessing legal compliance. Nevertheless, defining such thresholds is a challenge, since fairness requirements may vary depending on AI system´s clinical purpose, target populations, disease area, model types, fairness metrics, and associated risk levels (57). High-level recommendations in this topic must be addressed to operationalize such difficulties.

Likewise, the integration of users' (e.g., clinicians’) feedback mechanisms and performance metrics is highly recommended (58). Including post-deployment feedback loops, acting as real-time biases monitoring protocols is essential to robustly adjust and optimize AI systems and address biases during the AI system´s operational lifespan (Figure 2E) (32).

Conducting regular algorithmic audits is frequently addressed as a bias mitigation strategy at development stages to assess model's fairness (15). Additionally, performing audits during post-deployment stages is also required for monitoring bias, to uncover modeĺs underperformance in certain population groups, and for triggering the fine-tuning of the model performance over time as needed (37, 58).

Effective AI governance requires auditing standards and practices as transversal activities across the AI lifecycle, promoting transparency through detailed reporting on assessed AI models, audited datasets, test results, mitigation plans, actions taken, and system changes to ensure compliance (Figure 2E) (58, 59). Internal audits are required to map errors, assess representativeness, and make modifications based on thresholds and user feedback, facilitating the fair operationalization of AI without compromising accuracy, and ensuring technical, ethical and legal compliance (59, 60). Additionally, independent audits, performed by external auditors (third parties), both before and after clinical implementation approval, are also reported as a valuable strategy to ensure independent validation of fairness compliance, being crucial to enhance trustworthiness and transparency particularly at industry and societal level (3, 26).

Meeting AI Act and General Data Protection Regulation (GDPR) transparency and traceability requirements throughout the AI lifecycle is pivotal for audit purposes, enabling clear track of algorithmic decisions, identification of system failure and inequitable outcomes, and to determine responsible parties (3, 61). The incorporation of robust explainability features, comprehensive technical documentation and audit trails during the AI lifecycle will allow for regulators, authorities and auditing entities to interpret and trace AI outputs (Figure 2E) (61). By making the recommendations generated by AI opaque models more understandable, explainable AI (XAI) facilitates the identification of potential biases, supporting better algorithmic auditing and fostering accountability (Figure 2E) (62). Explainable AI (XAI) tools and models, like Shapley Additive Explanations (SHAP), Local Interpretable Model-agnostic Explanations (LIME) and Gradient-weighted Class Activation Mapping (Grad-CAM), are reported to enhance interpretability (63). Nevertheless, literature report limited progress in the incorporation of XAI methods into clinical workflows, due to its complex operationalization into governance frameworks, with future research being necessary to establish guidance for their interpretation, medical validation, and clinical adoption (62, 64). For instance, embedding user-friendly XAI tools is essential to promote fairness and transparency during human oversight and to build trust among stakeholders' involved in AI lifecycle, when considering a human-on-the-loop approach, thus propelling the responsible use of AI in post-deployment scenarios (49, 51, 63).

Thus, structured audit frameworks must be in place to assess algorithmic fairness performance and to apply correction measures (58).

Recommendations on how to structure AI audit frameworks have been developed (60, 65). These comprise several steps, including scoping, mapping vulnerabilities, collecting documentation, testing (e.g., errors analysis, adversarial and subgroup testing), reflection (e.g., risk mitigation strategies, recommendations and actions taken), and post-audit decisions (e.g., key findings report and re-audit) (60). Components related to model's technicality and functionality, such as input datasets used for model training and validity, design choices (e.g., code and features defined at pre-processing stages), algorithmic outcomes generated, and fairness validation, must be audited to determine the model performance and potential biased patterns (66). Despite these recommendations highlight the importance of technical, clinical, regulatory and ethical competences in the auditing team (60), harmonized guidance on the formal necessary audit roles is missing. Moreover, although regular audits should be conducted for high-risk AI systems during post-deployment (56), no formal requirements on auditing periodicity for internal and external audits have been defined.

Tools, such as fairness dashboards, bias assessment open-source toolkits (e.g., IBM's AI Fairness 360, Google's What-If Tool), algorithmic bias detection systems and audit logs, offer proactive fairness-aware mechanisms for auditing that must be part of the AI governance structure (50, 56, 64). Their integration in real-world workflows would support practitioners, developers and authorities during oversight routine regarding fairness without disrupting operations or overburdening the team (50). However, implementing auditing toolkits may entail financial and administrative costs, since 10 to 14% of development costs are already estimated for AI Act certification compliance (59).

The overarching goal of the auditing process is to enhance confidence and trust in the underlying system, possibly by formalizing this trust through an appropriate certification mechanism (65). Hence, to establish an ongoing auditing ecosystem, a cross-disciplinary team that combines a wide expertise ranging from clinicians, bioinformatics, data science, legal and ethical specialists must be engaged throughout the evaluation of AI systems. Previous studies emphasized the need for investing resources in workforce for the shared responsibility of monitoring high-risk AI systems for healthcare (60).

The MDR requires that AI-based medical devices obtain CE certification and regulatory approval from notified bodies before they can be marketed (17). With the AI Act in effect, manufacturers of high-risk systems must provide technical documentation and implement quality management systems (16). Contrary to traditional medical devices, the continuous learning nature of AI systems complicates regulatory approvals, creating potential bottlenecks for ongoing monitoring and posing synchronization challenges for AI regulators. This suggests that regulatory approval should be time-limited and periodically revisited, and subject to AI certifications to fairness updates based on audit feedback (Figure 2E) (15, 59). Additionally, regulatory incentives, through funding opportunities, formal audit frameworks and compliance certifications, were described as potential enablers to encourage developers and institutions to adopt AI ethical practices (46, 49, 67). An example of an initiative to certify algorithmic bias and fairness is the IEEE Ethics Certification Program for Autonomous and Intelligent Systems (49). The creation of certification bodies for labelling AI products for several parameters, namely data representativeness, applied bias mitigation strategies, among others, has been also proposed (68).

#### 4.2.2 Establishing governance frameworks and coordination mechanisms

AI governance is an essential piece to ensure that AI systems are designed, developed, deployed and operated in accordance with ethical principles and with the legal and societal requirements for transparency, accountability, security, and fairness (37).

By clearly defining stakeholder responsibilities, assessment criteria, escalation processes, and corrective measures throughout the AI lifecycle, AI governance supports the effective operationalization of the monitoring, auditing and oversight mechanisms described above.

A robust AI governance framework provides guidance, oversight mechanisms and tools throughout the AI lifecycle, contributing to strengthen trust among stakeholders and facilitating the adoption of the AI system (67). Nevertheless, current evidence suggests that such governance framework is not widely established (69).

Building into these findings and considerations, Figure 3 brings together the main components of institutional governance framework for the deployment of fair AI in healthcare, outlining the elements needed to support its coordinated implementation.

**Figure 3:**
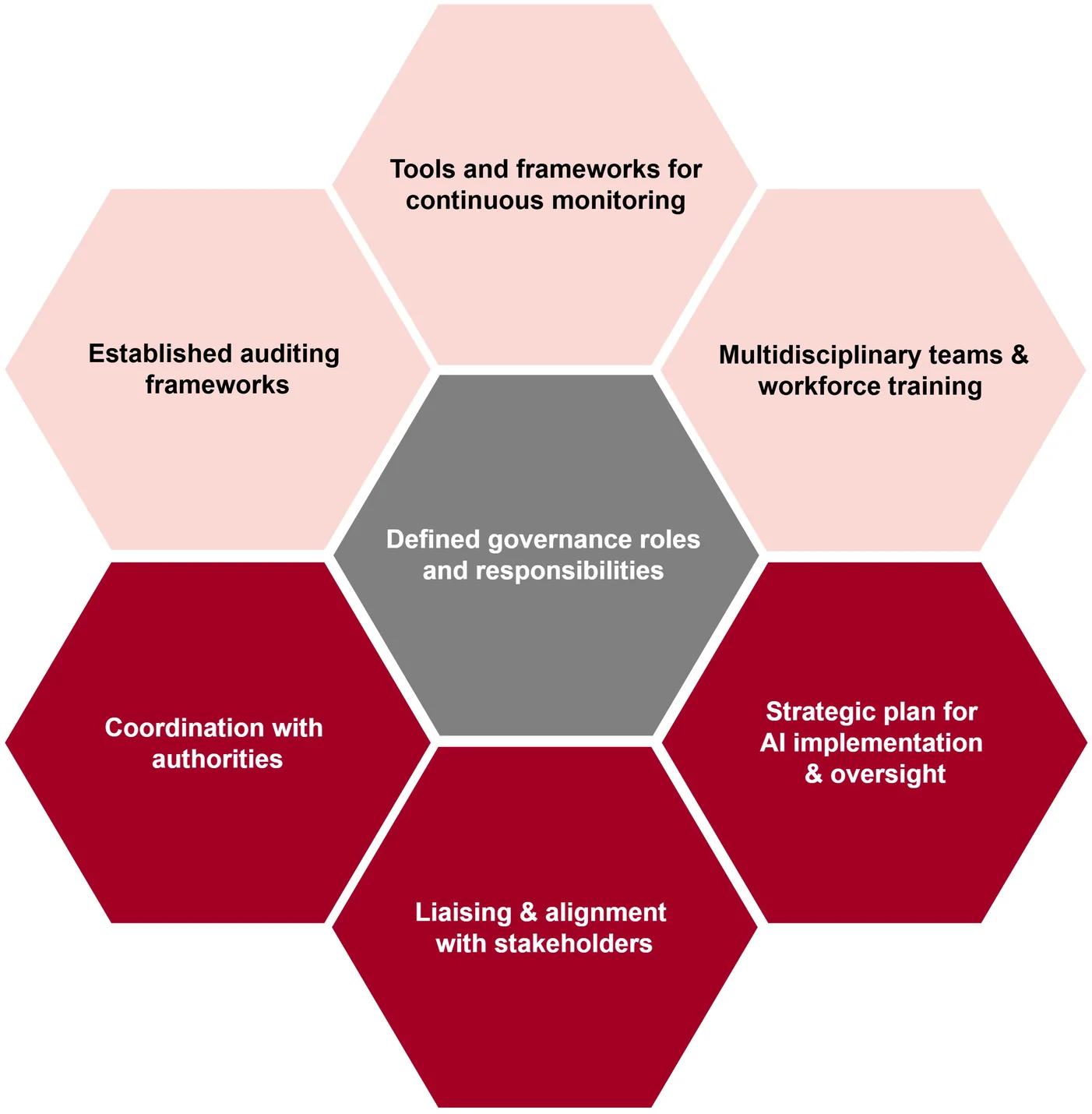
Core components of institutional AI governance for fair AI in healthcare.

Clearly defined governance roles and responsibilities are at the center of this governance framework (Figure 3). Multidisciplinary stakeholders (i.e., AI researchers, healthcare professionals, policymakers, and regulators) (70, 71) should be involved and share responsibility for setting fairness performance benchmarks, overseeing continuous monitoring, and enforcing regulatory and legal compliance in accordance with their expertise and interaction with the AI system (72). Moreover, AI governance structures should be closely integrated with data governance procedures (i.e., data management practices for training, tuning and testing methods for bias identification and mitigation) (39, 54), as part of a core operational and standardized methodology, involving stakeholders responsible for data collection and pre-processing tasks. This integration addresses the close interplay between AI governance, data quality, data representativeness, and algorithmic fairness (73).

Indeed, the involvement and collaboration of multidisciplinary stakeholders is a crucial component of a robust governance framework towards fairness (Figure 3), by enabling clear allocation of responsibilities and the integration of organizational, clinical, ethical, legal, and technical expertise across the AI lifecycle.

The involvement of healthcare practitioners and users throughout the AI lifecycle can support the consideration of different perspectives in system´s design and development, as well as in governance, policy and decision-making processes. Hence, promoting stakeholder continuous feedback and engagement, including after deployment, can foster fair and human-centred AI deployment (13).

Moreover, the institution's AI governance model should provide guidance for decision-making throughout all the AI lifecycle, defined continuous monitoring, audit frameworks and tools, robust risk management procedures, clear accountability frameworks, and mechanisms that ensure proactive mitigation of alterations that AI systems compliance may face upon advances in technology or alterations in the regulatory landscape (Figure 3) (37, 67, 72). This model ought to evolve interactively as regulatory, technological, ethical and clinical contexts change.

A strategic AI implementation and oversight plan should address hurdles such as alignment with regulatory requirements, and consider the institution's available resources, infrastructure, and tolerance to risk, all within a cohesive governance framework (27). Moreover, despite governance models should be institution-specific to allow for adaptability to the specific context and characteristics of the AI system, national high-level guidance on monitoring thresholds, compliance frameworks and overall governance procedures would be beneficial to support the consistent development of AI governance frameworks.

A survey across the WHO European Region provided a region-wide assessment of Member States' AI preparedness and how they are navigating this evolving field for healthcare. Only 54% of Member States' reported having one or more regulatory agencies responsible for assessing and approving AI systems in health but fewer had agencies tasked with post-deployment monitoring and surveillance of AI products (28). The findings demonstrated that AI governance strategies in healthcare remain uneven, with few Member States adopting health-specific strategies and many relying on cross-sectoral approaches, often managed by existing government agencies (28, 29).

Under the AI Act, national competent authorities, named market surveillance authorities, are central for regulation compliance and supervising high-risk AI systems at Member State level. They will be responsible for investigating risks, enforce corrective measures, perform continuous monitoring, and access documentation (e.g., datasets and code) to ensure compliance with the requirements of the AI Act. Also, these bodies will have the duty to report annually to the EC and other national authorities about their activities and identified risks (74). Currently, the European Commission (EC) is advancing a set of guidelines with instructions to support stakeholders' needs and compliance with the AI Act and relevant EU legislation (75).

Each EU Member State must designate one or more market surveillance authorities, based on national needs and sectoral responsibilities. When multiple authorities are appointed, a “Single Point of Contact” must be designated to liaise with the public, other Member States, and EU counterparts (74). This showcases the need to provide close coordination between the national AI surveillance authorities and the institutions developing AI systems (Figure 3). However, it remains unclear how this can be implemented in practice, especially in countries opting for a single cross-sector market surveillance authority. Also, the exact operational model (i.e., what will be supervised per sector, how the inspections will be prioritised, and how sector-specified will be evaluated) is still to be defined. Likewise, the “Single Point of Contact” list is still, in some cases, pending or incomplete.

### 4.3 Recommendations and future considerations for the deployment of fair AI for healthcare

Building on the findings discussed in the previous sections, recommendations were derived by translating the identified fairness-related gaps and the needs mapped from the core components of the governance framework into operational actions across the AI lifecycle. These recommendations are summarized in Table 2, identified with their relevant AI lifecycle stage(s), the key stakeholders involved in their operationalization, alongside proposed implementation actions.

**Table 2:** Recommendations to operationalize fairness in AI systems for healthcare (^a^All stages cover: design, development, clinical deployment and post-deployment monitoring; ^b^Multidisciplinary review committee cover: AI developers, data scientists; data curators, AI vendors/providers, ethicists, clinicians, healthcare institutions).

Governance levels	AI lifecycle stage	Stakeholders involved	Recommendation	Proposed solution
Institutional	Design and Development	AI developer Data curator Data scientist Ethicists Clinicians	Early stewardship and assessment of datasets	• Incorporation of multidisciplinary data governance committee responsible for dataset design, collection and data processing. • Incorporate measures to promote dataset balancing and adequate representativeness • Documentation of dataset characteristics and pre-processing techniques embedded in data curation.
	Development Clinical deployment Post-deployment monitoring	AI developer AI vendors/providers Data scientists Healthcare Institutions	Integration of fairness metrics for bias identification and mitigation during data and algorithmic bias assessment	• Definition and implementation of quantitative and performance fairness metrics throughout the AI lifecycle. • Harmonization of bias mitigation methods and fairness metrics. • Incorporation of fairness evaluation and mitigation during quality assessment and certification.
	All stages^a^	Auditors Notified Bodies AI developers AI manufacturers Healthcare Institutions Clinicians	Implementation of auditability mechanisms and governance models	• Defined audit frameworks, with formally outlined roles and frequency for internal and external audits. • Incorporation of technical reports for audit trails containing AI outputs and applied measures, XAI reports, along with relevant components (e.g., datasets and models) and previous evaluation results. • Establishment of multidisciplinary teams responsible for legal, ethical and clinical conformity assessment. • Flexible governance model that evolves according to context changes. • Definition of clear procedures, workflows and tools for monitoring fairness performance, and of auditing frameworks at institutional level.
National AI governance bodies	All stages^a^	National regulators National Notified Bodies National government bodies Multidisciplinary review committee^b^	Establish a national-level governance structure	• Definition of competent national surveillance entities, their designated roles (e.g., executive management, audit teams, technical experts, public relations officers, training teams, etc) and competencies. • Establish guidance to support fairness monitoring at AI developing national institutions, based on international level guidance. • Definition of fairness thresholds and national accountability frameworks (technical and legal), classification of misuses and errors. • Establishment of national enforcement frameworks, encompassing defined penalties and sanctions for non-compliance and dedicated adjudication bodies. • Development of strategic and budgetary guidance for sustainability. • Promotion of knowledge-sharing, liaising and cooperation between stakeholders, national authorities, European AI office and global AI regulatory bodies.
	Clinical deployment	National AI Auditors National regulators National Notified Bodies National government bodies Multidisciplinary review committee^b^	Establish national fairness-aware AI certification and conformity assessment harmonization	• Introduction of fairness-aware AI certification guidelines. • Incorporation of audit feedback on fairness evidence into AI certification updates. • Partnership between regulatory entities, researchers, institutions and manufacturers for fairness toolkits application and evolvement.
Supranational AI regulatory bodies	All stages^a^	Policy officers Legal officers Auditors AI specialists Data privacy officers	Establish and coordinate multi-stakeholders’ community	• Provide support to national AI governance bodies to create and establish fairness guidelines and best practices. • Creating a community of practice (CoP) and joint actions under their stewardship for continuous alignment. • Supporting harmonized technical, legal and ethical governance strategies and knowledge to establish fairness in AI systems.
	All stages^a^	Policy officers Legal officers Auditors AI specialists Data privacy officers	Establish a global level governance structure	• Institute mandatory national AI governance bodies. • Establish supranational benchmarks for AI fairness.

The proposed recommendations operationalize the governance needs identified in section 4.2 and extend these across different levels, namely institutional, national, and supranational. Through these levels, the recommendations address key actions that range from fairness assessment and mitigation, auditability and accountability, stakeholder involvement, to coordination plans.

The operationalization of these recommendations across the three governance levels demands for a sociotechnical strategy, addressing bias at multiple levels, by aligning technical measures with ethical, legal, organizational, clinical, and regulatory mechanisms to achieve equitable AI-driven healthcare (52, 70, 71, 76, 77). Such a strategy should draw on the capacity and organizational expertise of multidisciplinary stakeholders, institutions and relevant authorities according to each party's readiness, needs, and responsibilities according to their involvement throughout AI lifecycle stages.

Given this context and the rapidly evolving landscape of AI in healthcare, fairness workflows should be established across the AI lifecycle, by combining technical assessment with clearly defined organizational responsibilities. This should begin with the early assessment and stewardship of datasets, followed by the definition of fairness metrics, bias mitigation approaches, and harmonized methodologies for monitoring fairness. These actions should be attributed to multidisciplinary stakeholders, and clearly assigned according to their role in the lifecycle stage in which they intervene (70, 78).

Moreover, institutional governance bodies should provide mechanisms for continuous monitoring, auditing, and reporting, guarantying that the appropriate tools and procedures are in place to support the iterative adjustments needed to mitigate medium and long-term risks and susceptibilities in AI performance, such as those resulting from model drift and emerging biases within the clinical context (50, 79).

The literature also emphasizes shared accountability as a key factor for ensuring AI fairness throughout the AI lifecycle. Developers, deployers, clinicians, data scientists, data governance teams, and oversight bodies all should play complementary roles in identifying, mitigating, and monitoring bias (49, 70, 79). Examples of how these responsibilities may be distributed across stakeholders throughout the AI lifecycle have already been proposed in the literature (70, 71, 78, 79).

Likewise, auditing AI systems for healthcare is crucial both to comply with legal requirements and to foster trustworthiness in responsible AI. Audit regularity and architecture, namely its workflows, reporting procedures, and implications, should be defined according to real-world deployment needs and applicable legal norms (71). This calls for the involvement of governance bodies and several key players (e.g., auditors, notified bodies, developers, and clinicians) across all the lifecycle, specifically during clinical deployment and post-deployment monitoring (49, 71).

Therefore, the implementation of auditability mechanisms is central to operationalizing these responsibilities. Providing thorough technical documentation, with dataset characteristics, validation models, bias mitigation plans, XAI reports, and previous evaluation results, can support audit trails and clarify accountability across the AI lifecycle (70, 79). An example of a framework for fairness checks and supporting documentation along the AI lifecycle for review boards has been described in the literature (80).

While all the abovementioned mechanisms can be operationalized at institution level, their effective implementation depends on the establishment of a robust governance framework capable of supporting and coordinating fairness practices throughout the stages of the AI lifecycle (81). National governance bodies can provide guidance on standardized monitoring and auditing, shared accountability frameworks, fairness thresholds, and mechanisms for classifying errors. Moreover, the establishment of enforcement regimes, such as penalties for cases of non-compliance, may further support the operationalization of fairness (76, 77, 81).

In the EU scenario, a dual supervision approach, combining the AI Act's market surveillance for compliance auditing with different sector regulators evaluating distinct aspects namely purpose, post-market use and cybersecurity settings, may be crucial for regulating high-risk AI systems for healthcare. A lifecycle supervision model, led by a cross-functional unit, could assess AI fairness practices and ensure trustworthiness in real-world adaptation. This strategy would directly impact the operationalization of fairness practices under the EU regulatory frameworks, ensuring both compliance and ethical AI practices in healthcare.

Such operationalization at national level requires a harmonized AI regulatory landscape ensured by specialized supranational governance bodies (i.e., organization whose authority extends beyond a single nation) dedicated to supporting the monitoring and regulation of AI systems for healthcare (76, 77, 81, 82). At the supranational governance level, coordination becomes particularly important for the harmonization of technical, legal, and ethical approaches across national governance systems. Supranational bodies can provide such support through shared guidance and multistakeholder cooperation, promoting a greater consistency in how fairness is assessed and governed across jurisdictions.

## 5 Conclusions

This review identifies current practices and gaps towards the operationalization of trustworthy AI in healthcare, highlighting that this must be accompanied by equitable principles to guarantee fair healthcare treatment. The findings emphasize the importance of implementing fairness-aware practices, data governance and management frameworks, and robust oversight mechanisms throughout the AI lifecycle to identify and mitigate potential sources of bias. The success of implementing such measures will depend on the coordinated efforts of a solid governance structure at institutional level, guided by national and supranational governance bodies to facilitate the harmonization of bias mitigation measures, consistent governance frameworks and the continuous monitoring of AI systems for health applications. Based on study's findings, a set of recommendations to operationalize fairness in AI systems for healthcare is proposed. The proposed recommendations are grounded in the governance framework fairness-related gaps and the needs, being thus particularly relevant for promoting the responsible and equitable integration of AI in healthcare practice. Nevertheless, the feasibility and practical operationalization of these recommendations require future assessment and validation in real-world healthcare settings. Hence, aligning these practices with future dedicated actions and engagement with multidisciplinary stakeholders will be crucial for workforce training, building trust and ensuring the equal benefits that AI might carry for healthcare and society.
