# Supplementary material for: Operationalizing fair AI in healthcare: a scoping review of bias mitigation, monitoring, auditing, and governance strategies
Source: Front Digit Health. 2026 Oct 13;8:1888744. doi: 10.3389/fdgth.2026.1888744 (PMC13711374; doi:10.3389/fdgth.2026.1888744)
Supplement: Supplementary file 1 [file Datasheet1.docx]

Supplementary Material

**Supplementary Figure 1.** AI lifecycle framework applied in this scoping review. The four high-level stages – design, development, deployment and post-deployment, adopted for categorizing the evidence along the AI lifecycle are shown, together with illustrative processes encompassed within each stage and cross-cutting activities.

**Supplementary Table 1.** The selected institutional sources and focus area for the grey literature.

| Institution | Type of source | Focus Area |
| --- | --- | --- |
| European Commission (EC) and Parliament | Regulations, official documents and informative materials | AI Act, MDR and market surveillance |
| European Medicine Agency (EMA) | Reports, scientific guidelines, opinion materials, referral documents, regulatory and procedural guidelines | AI and medical devices lifecycle |
| Food and Drug Administration (FDA) | Guidance and recommendations | AI lifecycle and bias monitoring |
| World Health Organization (WHO) | Publications, reports and guidance | Artificial intelligence, governance, ethical, health, Europe Region |
